# Long-term effect of sitagliptin on endothelial function in type 2 diabetes: a sub-analysis of the PROLOGUE study

**DOI:** 10.1186/s12933-016-0438-x

**Published:** 2016-09-13

**Authors:** Tatsuya Maruhashi, Yukihito Higashi, Yasuki Kihara, Hirotsugu Yamada, Masataka Sata, Shinichiro Ueda, Masato Odawara, Yasuo Terauchi, Kazuoki Dai, Jun Ohno, Masato Iida, Hiroaki Sano, Hirofumi Tomiyama, Teruo Inoue, Atsushi Tanaka, Toyoaki Murohara, Koichi Node, for the PROLOGUE Study Investigators

**Affiliations:** 1 Department of Cardiovascular Medicine, Graduate School of Biomedical and Health Sciences, Hiroshima University, 1-2-3 Kasumi, Minami-ku, Hiroshima, 734-8551 Japan; 2 Division of Regeneration and Medicine, Medical Center for Translational and Clinical Research, Hiroshima University Hospital, 1-2-3 Kasumi, Minami-ku, Hiroshima, 734-8551 Japan; 3 Department of Cardiovascular Regeneration and Medicine, Research Institute for Radiation Biology and Medicine, Hiroshima University, 1-2-3 Kasumi, Minami-ku, Hiroshima, 734-8551 Japan; 4 Department of Cardiovascular Medicine, Tokushima University Hospital, 2-50-1 Kuramoto-cho, Tokushima, 770-0042 Japan; 5 Department of Cardiovascular Medicine, Institute of Biomedical Sciences, Tokushima University Graduate School, 3-18-15 Kuramoto-cho, Tokushima, 770-0042 Japan; 6 Department of Clinical Pharmacology and Therapeutics, University of the Ryukyus, 207 Uehara, Nishihara-cho, Okinawa 903-0215 Japan; 7 Department of Diabetes, Endocrinology, Metabolism and Rheumatology, Tokyo Medical University, 6-1-1 Nishishinjuku, Shinjuku-ku, Tokyo, 160-0023 Japan; 8 Department of Endocrinology and Metabolism, Yokohama City University, 3-9 Fukuura, Kanazawa-ku, Yokohama, 236-0004 Japan; 9 Department of Cardiology, Hiroshima City Hospital, 7-33 Motomachi, Naka-ku, Hiroshima, 730-0011 Japan; 10 Department of Cardiology, Tsushima Municipal Hospital, 3-73 Tachibana-cho, Tsushima, 496-0038 Japan; 11 Department of Cardiology, Mitsubishi Nagoya Hospital, 7-8 Sotodoi-cho, Atsuta-ku, Nagoya, 456-0013 Japan; 12 Department of Cardiology, Nagoya Ekisaikai Hospital, 4-66, Syounen-cho, Nakagawa-ku, Nagoya, 454-0854 Japan; 13 Department of Cardiology, Tokyo Medical University, 6-7-1, Nishishinjuku, Shinjuku-ku, Tokyo, 160-0023 Japan; 14 Department of Cardiovascular Medicine, Dokkyo Medical University, 880 Kitakobayashi, Mibumachi, Shimotsuga-gun, Tochigi 321-0293 Japan; 15 Department of Cardiovascular Medicine, Saga University, 5-1-1, Nabeshima, Saga, 849-0937 Japan; 16 Department of Cardiology, Nagoya University Graduate School of Medicine, 65 Tsurumai-cho, Shouwa-ku, Nagoya, 466-0065 Japan

**Keywords:** Dipeptidyl peptidase 4 inhibitor, Flow-mediated vasodilation, Type 2 diabetes

## Abstract

**Background:**

As a sub-analysis of the PROLOGUE study, we evaluated the long-term effect of sitagliptin, a dipeptidyl peptidase 4 inhibitor, on endothelial function in the conduit brachial artery in patients with type 2 diabetes.

**Methods:**

In the PROLOGUE study, patients were randomly assigned to either add-on sitagliptin treatment (sitagliptin group) or continued conventional antihyperglycemic treatment (conventional group). Among the 463 participants in the PROLOGUE study, FMD was measured in 17 patients in the sitagliptin group and 18 patients in the conventional group at the beginning and after 12 and 24 months of treatment.

**Results:**

HbA1c levels were significantly decreased after 12 and 24 months of treatment compared to baseline values in both groups (7.0 ± 0.4 vs. 6.6 ± 0.3 and 6.6 ± 0.4 % in the sitagliptin group; 7.0 ± 0.6 vs. 6.6 ± 0.7 and 6.6 ± 0.7 % in the conventional group; P < 0.05, respectively). There was no significant difference between FMD values at baseline and after 12 and 24 months in the sitagliptin group (4.3 ± 2.6 vs. 4.4 ± 2.1 and 4.4 ± 2.3 %, P = 1.0, respectively). Although FMD had a tendency to increase from 4.3 ± 2.4 % at baseline to 5.2 ± 1.9 % after 12 months and 5.1 ± 2.2 % after 24 months in the conventional group, there was no significant difference between FMD values at baseline and after 12 and 24 months (P = 0.36 and 0.33, respectively).

**Conclusions:**

Add-on sitagliptin to conventional antihyperglycemic drugs in patients with type 2 diabetes did not alter endothelial function in the conduit brachial artery measured by FMD during a 2-year study period. Sitagliptin may be used without concern for an adverse effect on endothelial function in patients with type 2 diabetes.

*Trial registration:* University hospital Medical Information Network (UMIN) Center: ID UMIN000004490

**Electronic supplementary material:**

The online version of this article (doi:10.1186/s12933-016-0438-x) contains supplementary material, which is available to authorized users.

## Background

Endothelial dysfunction is the initial step in the pathogenesis of atherosclerosis and plays an important role in the development of this condition [1, 2]. In addition, it has been shown that endothelial function is an independent predictor of cardiovascular events [3]. Type 2 diabetes, an important risk factor for cardiovascular disease, is associated with endothelial dysfunction [4, 5]. Several investigators have reported that lifestyle modification and pharmacological therapy, including antihyperglycemic agents, improve endothelial function in patients with type 2 diabetes [6–9]. These findings suggest that endothelial dysfunction is reversible and can be restored through an appropriate intervention in patients with type 2 diabetes.

An orally administered dipeptidyl peptidase 4 (DPP-4) inhibitor is now available for treatment of type 2 diabetes. The DPP-4 inhibitor prolongs the action of incretin hormones by inhibition of their breakdown and improves glycemic control through incretin hormone-induced decrease in glucagon levels and increase in endogenous insulin secretion in patients with type 2 diabetes. The relationship between treatment with a DPP-4 inhibitor and endothelial function in patients with type 2 diabetes has been evaluated [10–13]. Sitagliptin, a DPP-4 inhibitor, has been demonstrated to significantly improve microvascular endothelial function assessed by the reactive hyperemia peripheral arterial tonometry index after 6 months in uncontrolled diabetic patients with coronary heart disease [10]. As for the relationship between a DPP-4 inhibitor and endothelial function in the conduit brachial artery assessed by flow-mediated vasodilation (FMD), a previous short-term study demonstrated that 6-week treatment with DPP-4 inhibitors, including sitagliptin and alogliptin, attenuated FMD [11], whereas other previous studies demonstrated that 12-week treatment with sitagliptin improved FMD in patients with type 2 diabetes [12, 13]. However, the long-term effect of a DPP-4 inhibitor on FMD in patients with type 2 diabetes remains unclear.

The PROLOGUE study was a prospective multicenter study conducted to evaluate the inhibitory effect of a DPP-4 inhibitor on progression of atherosclerosis based on carotid artery intima-media thickness (IMT) assessed by ultrasonography over a 2-year follow-up period [14]. In that study, FMD in the brachial artery was also measured in some of the subjects. Therefore, we carried out the present study as a sub-analysis of the PROLOGUE study to evaluate the long-term effect of a DPP-4 inhibitor on endothelial function assessed by FMD in the brachial artery in patients with type 2 diabetes.

## Methods

### Study design and patients

The rationale and design of the PROLOGUE study (University Hospital Medical Information Network Center: ID 000004490) have been described previously [15]. In brief, the PROLOGUE study was a multicenter, prospective, randomized, open-label trial and blinded-endpoint trial carried out with the participation of 48 Japanese institutions. Eligible patients were at least 30 years of age and who had type 2 diabetes with HbA1c level of 6.2–9.4 % despite conventional treatment with diet, exercise and/or pharmacologic therapy with oral antihyperglycemic agents (except incretin-related therapy) for more than 3 months. Patients who had taken a DPP-4 inhibitor, glucagon-like peptide-1 (GLP-1) analogs, or insulin before randomization were excluded. Other exclusion criteria are described elsewhere [15].

Between June 2011 and September 2012, a total of 463 patients with type 2 diabetes were enrolled and randomly assigned in a 1:1 ratio to either add-on sitagliptin treatment (sitagliptin group: n = 232) or conventional antihyperglycemic treatment (conventional group: n = 231). The treatment randomization was conducted on basis of the age, gender, use of statins, pre-treatment diabetic type (non-pharmacological or pharmacological treatment), HbA1c (<7 or ≥7 %), office systolic blood pressure (<135 or ≥135 mm Hg), and maximum IMT (<1.0 or ≥1.0 mm) [15]. All patients were treated with the aim of achieving a targeted HbA1c level less than 6.2 % or fasting plasma glucose level less than 110 mg/dL during the study period. Treatment of patients in the sitagliptin group was initially started with sitagliptin at a dose of 50 mg daily. If further glycemic intervention was necessary, the dose of sitagliptin was increased up to 100 mg daily within 3 months, and conventional antihyperglycemic agents other than DPP-4 inhibitors, GLP-1 analogs and/or insulin were added. If further glycemic intervention was necessary in patients in the conventional group, antihyperglycemic agents other than DPP-4 inhibitors, GLP-1 analogs and/or insulin were added. All of the patients were followed up annually for 2 years until September 2014.

In the PROLOGUE study, the primary endpoint was the change in mean common carotid artery-IMT at 24 months after treatment. Carotid ultrasound examinations were performed at the beginning of treatment and after 12 and 24 months of treatment. The secondary outcomes included changes in FMD in the brachial artery after 12 and 24 months of treatment [15]. In some of the participating institutions, FMD in the brachial artery was also measured as an optional examination. Of a total of 463 patients, serial measurement of FMD was performed in 17 patients in the sitagliptin group and 18 patients in the conventional group at the beginning and after 12 and 24 months of treatment. The data for these 35 patients from 4 institutions were analyzed in the present study. This sub-study is a pre-specified analysis. The ethical committees of the participating institutions approved the study protocol. Written informed consent for participation in the study was obtained from all subjects.

### Study protocol

All studies were performed in the morning, after overnight fasting, in a quiet, dark, and air-conditioned room (constant temperature of 22–25 °C). The subjects were kept in the supine position throughout the study. A 23-gauge polyethylene catheter was inserted into the left deep antecubital vein to obtain blood samples. The vascular response to reactive hyperemia in the brachial artery was used for the assessment of endothelium-dependent FMD. The observers were blind to the form of examination.

### Measurement of FMD

The same protocol for measurement of FMD in the brachial artery was used in the study. FMD was measured using the same ultrasound instrument specialized for FMD measurements in all institutions. A high-resolution linear artery transducer was coupled to computer-assisted analysis software (UNEXEF18G, UNEX Co, Nagoya, Japan) that used an automated edge detection system for measurement of brachial artery diameter. A blood pressure cuff was placed around the forearm. The brachial artery was scanned longitudinally 5–10 cm above the elbow. When the clearest B-mode image of the anterior and posterior intimal interfaces between the lumen and vessel wall was obtained, the transducer was held at the same point throughout the scan by a special probe holder (UNEX Co) to ensure consistency of the image. Depth and gain setting were set to optimize the images of the arterial lumen wall interface. When the tracking gate was placed on the intima, the artery diameter was automatically tracked, and the waveform of diameter changes over the cardiac cycle was displayed in real time using the FMD mode of the tracking system. This allowed the ultrasound images to be optimized at the start of the scan and the transducer position to be adjusted immediately for optimal tracking performance throughout the scan. Pulsed Doppler flow was assessed at baseline and during peak hyperemic flow, which was confirmed to occur within 15 s after cuff deflation. Blood flow velocity was calculated from the color Doppler data and was displayed as a waveform in real time. The baseline longitudinal image of the artery was acquired for 30 s, and then the blood pressure cuff was inflated to 50 mm Hg above systolic pressure for 5 min. The longitudinal image of the artery was recorded continuously until 5 min after cuff deflation. Pulsed Doppler velocity signals were obtained for 20 s at baseline and for 10 s immediately after cuff deflation. Changes in brachial artery diameter were immediately expressed as percentage change relative to the vessel diameter before cuff inflation. FMD was automatically calculated as the percentage change in peak vessel diameter from the baseline value. Percentage of FMD [(Peak diameter − Baseline diameter)/Baseline diameter] was used for analysis. Blood flow volume was calculated by multiplying the Doppler flow velocity (corrected for the angle) by heart rate and vessel cross-sectional area (−r^2^). Reactive hyperemia was calculated as the maximum percentage increase in flow after cuff deflation compared with baseline flow. Inter- and intra-coefficients of variation for the brachial artery diameter were 1.6 and 1.4 %, respectively.

### Statistical analysis

Results are presented as mean ± SD. All reported probability values were 2-sided, and a probability value of <0.05 was considered statistically significant. Categorical variables were compared by means of the Chi square test. We compared mean values of continuous variables between the 2 groups by unpaired Student’s *t* test. Differences in mean values of continuous variables between baseline, 12 and 24 months were compared by paired Student’s *t* test with Bonferroni’s correction. The data were processed using the software package Stata version 9 (Stata Co., College Station, Texas, USA).

## Results

### Baseline clinical characteristics

Table 1 shows the baseline clinical characteristics of all patients and the effects of each treatment on baseline parameters in the sitagliptin group and conventional group. Of the 35 patients, 20 (57.1 %) were men and 15 (42.9 %) were women. Twenty-six (74.3 %) had hypertension, 25 (71.4 %) had dyslipidemia, 5 (19.2 %) were current smokers, 18 (51.4 %) had coronary heart disease, and 3 (8.5 %) had cerebrovascular disease. The mean fasting plasma glucose level was 7.04 ± 1.11 mmol/L and the mean HbA1c level was 7.0 ± 0.5 %. The mean value of FMD was 4.3 ± 2.4 %. There was no significant difference in any of the variables except the prevalence of current smokers between the two groups. Although serum levels of creatinine and lipids did not significantly change during the treatment period, systolic blood pressure was significantly higher after 24 months in the sitagliptin group than in the conventional group.

**Table 1:** Clinical characteristics of the subjects

Variables	All (n = 35)	Conventional group (n = 18)			Sitagliptin group (n = 17)		
	0 month	0 month	12 months	24 months	0 month	12 months	24 months
Age, y	66.5 ± 8.9	64.1 ± 10.3			69.1 ± 6.5		
Male, n (%)	20 (57.1)	11 (61.1)			9 (52.9)		
Body mass index, kg/m^2^	27.0 ± 4.2	27.2 ± 5.0	27.1 ± 4.9	27.0 ± 4.8	26.8 ± 3.3	26.9 ± 3.3	26.5 ± 3.0
Systolic blood pressure, mm Hg	128.0 ± 13.1	127.2 ± 14.0	129.5 ± 15.3	123.6 ± 12.5	136.9 ± 16.7	138.6 ± 15.6	133.2 ± 13.3*
Diastolic blood pressure, mm Hg	72.8 ± 10.1	78.8 ± 10.6	75.4 ± 10.9	72.4 ± 10.2	79.8 ± 8.9	78.5 ± 8.8	76.5 ± 8.8
Heart rate, bpm	67.3 ± 9.5	67.1 ± 8.6	67.7 ± 10.3	67.9 ± 8.5	67.2 ± 9.6	65.6 ± 10.7	68.0 ± 12.9
Creatinine, μmol/L	72.0 ± 19.5	71.8 ± 19.3	70.5 ± 19.6	79.5 ± 24.6	72.2 ± 20.2	72.2 ± 22.7	76.8 ± 24.3
Total cholesterol, mmol/L	4.66 ± 0.75	4.74 ± 0.87	4.95 ± 1.19	4.72 ± 0.98	4.57 ± 0.60	4.57 ± 0.78	4.60 ± 0.69
Triglycerides, mmol/L	1.38 ± 0.48	1.49 ± 0.47	1.55 ± 0.53	1.19 ± 0.31	1.26 ± 0.47	1.26 ± 0.65	1.43 ± 0.97
HDL cholesterol, mmol/L	1.42 ± 0.37	1.38 ± 0.45	1.44 ± 0.40	1.45 ± 0.37	1.47 ± 0.27	1.46 ± 0.33	1.47 ± 0.34
Glucose, mmol/L	7.04 ± 1.11	7.03 ± 1.04	7.14 ± 1.46	6.66 ± 1.67	7.05 ± 1.21	6.66 ± 1.27	6.48 ± 0.81
HbA1c, %	7.0 ± 0.5	7.0 ± 0.6	6.6 ± 0.7	6.6 ± 0.7	7.0 ± 0.4	6.6 ± 0.3	6.6 ± 0.4
Brachial artery diameter, mm	4.08 ± 0.55	4.05 ± 0.60	4.02 ± 0.60	3.98 ± 0.58	4.11 ± 0.52	4.14 ± 0.59	4.19 ± 0.67
Current smoker, n (%)	5 (19.2)	5 (41.7)			0 (0)*		
*Complications*							
Hypertension, n (%)	26 (74.3)	13 (72.2)			13 (76.5)		
Dyslipidemia, n (%)	25 (71.4)	11 (61.1)			14 (82.4)		
Coronary heart disease, n (%)	18 (51.4)	8 (44.4)			10 (58.8)		
Cerebrovascular disease, n (%)	3 (8.5)	2 (11.1)			1 (5.9)		
*Antidiabetic drugs*							
Sulfonylurea, n (%)	10 (28.6)	5 (27.8)	6 (33.0)	6 (33.0)	5 (29.4)	3 (17.6)	3 (17.6)
Metformin, n (%)	9 (25.7)	5 (27.8)	7 (38.9)	7 (38.9)	4 (23.5)	5 (29.4)	5 (29.4)
α-Glucosidase inhibitor, n (%)	13 (37.1)	4 (22.2)	8 (44.4)	8 (44.4)	9 (52.9)	6 (35.3)	6 (35.3)
Pioglitazone, n (%)	4 (11.4)	2 (11.1)	3 (16.7)	3 (16.7)	2 (11.8)	1 (5.9)	1 (5.9)
Glinide, n (%)	1 (2.9)	0 (0)	1 (5.6)	1 (5.6)	1 (5.9)	0 (0)	0 (0)
*Antihyperlipidemic drugs*							
Statin, n (%)	21 (60.0)	8 (44.4)	8 (44.4)	9 (50.0)	13 (76.4)	12 (70.1)	12 (70.1)
Fibrate, n (%)	2 (5.7)	2 (11.1)	2 (11.1)	2 (11.1)	0 (0)	0 (0)	0 (0)
Eicosapentaenoic acid, n (%)	2 (5.7)	1 (5.6)	1 (5.6)	1 (5.6)	1 (5.9)	1 (5.9)	1 (5.9)
Ezetimibe, n (%)	1 (2.9)	0 (0)	0 (0)	0 (0)	1 (5.9)	1 (5.9)	1 (5.9)
*Antihypertensive drugs*							
Calcium channel blocker, n (%)	21 (60.0)	11 (61.1)	11 (61.1)	11 (61.1)	10 (58.8)	10 (58.8)	10 (58.8)
ARB, n (%)	20 (57.1)	11 (61.1)	11 (61.1)	11 (61.1)	9 (52.9)	10 (58.8)	10 (58.8)
ACE inhibitor, n (%)	5 (14.2)	3 (16.7)	3 (16.7)	3 (16.7)	2 (11.8)	1 (5.9)	1 (5.9)
Diuretic, n (%)	8 (22.9)	3 (16.7)	3 (16.7)	4 (22.2)	5 (29.4)	5 (29.4)	5 (29.4)
Beta-blocker, n (%)	8 (22.9)	4 (22.2)	4 (22.2)	4 (22.2)	4 (23.5)	5 (29.4)	5 (29.4)
*Others*							
Antiplatelet agent, n (%)	18 (51.4)	7 (38.9)	7 (38.9)	7 (38.9)	11 (64.7)	12 (70.6)	12 (70.6)

*HDL* high-density lipoprotein; *ARB* angiotensin receptor blockers; *ACE* angiotensin converting enzyme

* P < 0.05 vs. control group

### Glycemic control

**Fig. 1:**
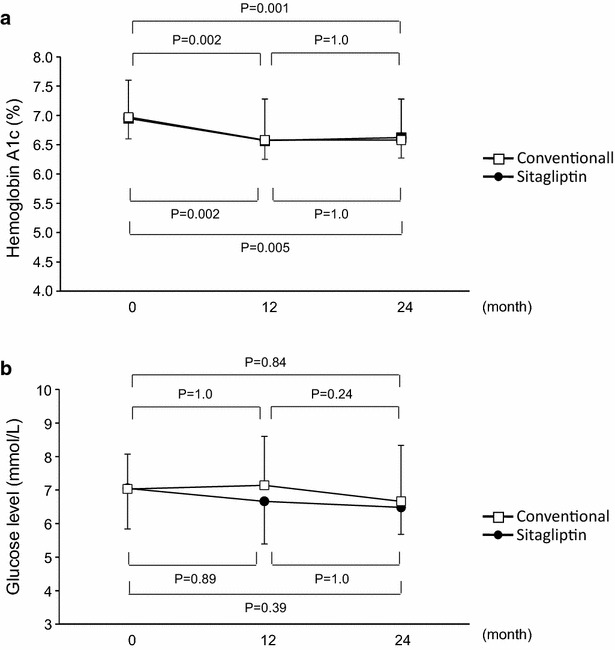
Line graphs show hemoglobin A1c level (**a**) and fasting glucose level (**b**) at each study visit in the sitagliptin group and conventional group

HbA1c and fasting plasma glucose levels were similar at baseline between the two groups. HbA1c levels were significantly decreased after 12 and 24 months of treatment compared to baseline values in both groups (7.0 ± 0.4 vs. 6.6 ± 0.3 and 6.6 ± 0.4 % in the sitagliptin group; 7.0 ± 0.6 vs. 6.6 ± 0.7 and 6.6 ± 0.7 % in the conventional group; P < 0.05, respectively, Fig. 1a). No significant difference in fasting plasma glucose level was observed during the study period in either group (Fig. 1b).

### Endothelial function

**Fig. 2:**
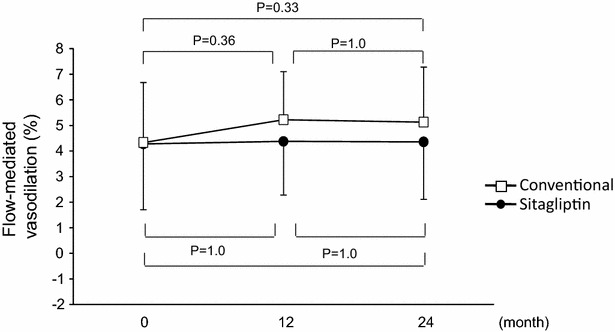
Line graphs show flow-mediated vasodilation at each study visit in the sitagliptin group and conventional group

Effects of glycemic intervention on FMD at baseline and after 12 and 24 months of treatment in the sitagliptin group and conventional group are shown in Fig. 2. FMD values were similar at baseline in the two groups. There was no significant difference between FMD values at baseline and after 12 and 24 months in the sitagliptin group (4.3 ± 2.6 vs. 4.4 ± 2.1 and 4.4 ± 2.3 %, P = 1.0, respectively). Although FMD rose from 4.3 ± 2.4 % at baseline to 5.2 ± 1.9 % after 12 months and 5.1 ± 2.2 % after 24 months in the conventional group, there was no significant difference between FMD values at baseline and after 12 and 24 months (P = 0.36 and 0.33, respectively). There was no significant difference between the two groups in FMD after 12 and 24 months (P = 0.22 and 0.31, respectively).

## Discussion

In the present study, similar degrees of improvement in glycemic control were achieved in the sitagliptin group and the conventional group. The present study demonstrated that the addition of sitagliptin to usual care in patients with type 2 diabetes did not alter endothelial function assessed by FMD in the conduit brachial artery over a 2-year study period.

In the present study, patients who had taken a DPP-4 inhibitor, GLP-1 analogs, or insulin before randomization were excluded. Moreover, additional use of incretin-related antihyperglycemic agents and insulin for further glycemic intervention was inhibited in the conventional group during the study period according to the study protocol. Therefore, the control treatment did not mask any true effect of sitagliptin in this study.

Short-term effects of treatment with sitagliptin on FMD have been controversially reported [11–13]. Ayaori et al. [11] demonstrated that 6-week sitagliptin therapy significantly attenuated FMD despite improved diabetic status, whereas two other previous studies demonstrated that 12-week sitagliptin therapy significantly improved FMD [12, 13], suggesting that at least 12 weeks of treatment with sitagliptin is necessary for improvement of endothelial function. However, the long-term effect of sitagliptin therapy on FMD has remained unclear. In the present study, we demonstrated that FMD was not altered after 12 and 24 months. FMD was maintained at a similar level during the study period by treatment with sitagliptin in patients with type 2 diabetes. Recently, a cardiovascular safety concern regarding the long-term use of some antihyperglycemic agents has been raised [16, 17]. Therefore, new antihyperglycemic agents are required not only to show glucose-lowering ability but also to be not associated with increases in major adverse cardiovascular events [18]. A cardiovascular effect of sitagliptin has been shown in experimental and clinical studies [19, 20]. In an experimental model, it was demonstrated that sitagliptin can reduce the area of atherosclerotic lesions, possibly by regulating the AMPK and MAPK pathways and then reducing leukocyte-endothelial cell interaction and inflammation reactions [19]. Moreover, sitagliptin treatment has neutral effects on left ventricular diastolic function in diabetic patients [20]. A recent study demonstrated that adding sitagliptin to usual care in patients with both type 2 diabetes and established cardiovascular disease did not increase the risk of major adverse cardiovascular events or hospitalization for heart failure during a median follow-up period of 3.0 years [21]. In the secondary analysis of the study, it was demonstrated that sitagliptin does not affect the risk of hospitalization for heart failure in patients with type 2 diabetes, both overall and among high-risk patient subgroups [22]. These results are supported by our finding that 2-year add-on sitagliptin therapy was not associated with impairment of endothelial function in the conduit brachial artery assessed by FMD, an independent predictor of cardiovascular events.

In the conventional group, FMD increased, but not significantly, from 4.3 ± 2.4 % at baseline to 5.2 ± 1.9 % after 12 months and 5.1 ± 2.2 % after 24 months. In accordance with the study protocol, the use of antihyperglycemic agents other than DPP-4 inhibitors, GLP-1 analogs and/or insulin was encouraged as required, with the aim of achieving the target HbA1c level in the conventional group during the study period. Several studies have shown that some antihyperglycemic agents have beneficial effects on endothelial function. Treatment with metformin, pioglitazone, or an α-glucosidase inhibitor in patients with type 2 diabetes has been demonstrated to improve endothelial function assessed by FMD [13, 23–26]. Patients in the conventional group received additional antihyperglycemic agents, including metformin, α-glucosidase inhibitor and pioglitazone, instead of sitagliptin added in the sitagliptin group, to achieve the target HbA1c level. The addition of these antihyperglycemic agents might have contributed to the increasing tendency in FMD in the conventional group.

### Limitations

A major limitation of this study is a small sample size. The present study was a sub-analysis of the PROLOGUE study, and the number of study subjects was relatively small. Unfortunately, there is no sample size for power calculation since FMD was a voluntary measurement parameter in the PROLOGUE trial, and this may be underpowered. Further studies enrolling a large number of subjects are needed to confirm the long-term effect of a DPP-4 inhibitor on endothelial function in patients with type 2 diabetes.

## Conclusions

Adding sitagliptin to usual care in patients with type 2 diabetes did not alter endothelial function in the conduit brachial artery measured by FMD during a 2-year study period. Sitagliptin may be used in patients with type 2 diabetes without concern for an adverse effect on endothelial function (Additional file 1).

## Additional file

10.1186/s12933-016-0438-x All dataset supporting the conclusions of this article.

## Abbreviations

DPP-4: dipeptidyl peptidase 4
FMD: flow-mediated vasodilation
GLP-1: glicagon-like peptide-1
IMT: intima-media thickness
